# Expression analysis of mitotic spindle checkpoint genes in breast carcinoma: role of *NDC80/HEC1 *in early breast tumorigenicity, and a two-gene signature for aneuploidy

**DOI:** 10.1186/1476-4598-10-23

**Published:** 2011-02-27

**Authors:** Ivan Bièche, Sophie Vacher, François Lallemand, Sengül Tozlu-Kara, Hind Bennani, Michèle Beuzelin, Keltouma Driouch, Etienne Rouleau, Florence Lerebours, Hugues Ripoche, Géraldine Cizeron-Clairac, Frédérique Spyratos, Rosette Lidereau

**Affiliations:** 1 INSERM U735, St-Cloud, F-92210, France. Institut Curie Hôpital René Huguenin, FNCLCC, St-Cloud, F-92210, France; 2 INSERM U745, Université Paris Descartes, Faculté des Sciences Pharmaceutiques et Biologiques, Paris, F-75006, France; 3 CNRS UMR 8200, Institut Gustave-Roussy, Villejuif, F-94805, France

## Abstract

**Background:**

Aneuploidy and chromosomal instability (CIN) are common abnormalities in human cancer. Alterations of the mitotic spindle checkpoint are likely to contribute to these phenotypes, but little is known about somatic alterations of mitotic spindle checkpoint genes in breast cancer.

**Methods:**

To obtain further insight into the molecular mechanisms underlying aneuploidy in breast cancer, we used real-time quantitative RT-PCR to quantify the mRNA expression of 76 selected mitotic spindle checkpoint genes in a large panel of breast tumor samples.

**Results:**

The expression of 49 (64.5%) of the 76 genes was significantly dysregulated in breast tumors compared to normal breast tissues: 40 genes were upregulated and 9 were downregulated. Most of these changes in gene expression during malignant transformation were observed in epithelial cells.

Alterations of nine of these genes, and particularly *NDC80*, were also detected in benign breast tumors, indicating that they may be involved in pre-neoplastic processes.

We also identified a two-gene expression signature (*PLK1 *+ *AURKA*) which discriminated between DNA aneuploid and DNA diploid breast tumor samples. Interestingly, some DNA tetraploid tumor samples failed to cluster with DNA aneuploid breast tumors.

**Conclusion:**

This study confirms the importance of previously characterized genes and identifies novel candidate genes that could be activated for aneuploidy to occur. Further functional analyses are required to clearly confirm the role of these new identified genes in the molecular mechanisms involved in breast cancer aneuploidy. The novel genes identified here, and/or the two-gene expression signature, might serve as diagnostic or prognostic markers and form the basis for novel therapeutic strategies.

## Introduction

A very large proportion of cancers consist of cells with an abnormal chromosome content, a feature known as aneuploidy [1]. Aneuploidy is often associated with chromosomal instability (CIN), a condition in which cancer cells show a high rate of chromosomal gain and loss compared with normal cells.

The mechanisms underlying CIN, although poorly understood, are likely to include defects in the mitotic machinery used to segregate duplicated chromosomes between daughter cells [2]. Mounting evidence points to the mitotic spindle checkpoint as the point of failure in CIN. The normal function of the spindle checkpoint is to ensure that all chromosomes are correctly aligned in metaphase cells and properly attached to the mitotic spindle before chromosome separation can proceed. Like other phenotypes characteristic of cancer, it was first thought that nucleotide mutations in genes that control chromosome stability were responsible for CIN. However, somatic point mutations in mitotic-spindle-checkpoint genes, including *MAD1, BUB1 *and *BUBR1/BUB1B*, are infrequent [3]. One possible explanation for this paradox is that mitotic-spindle-checkpoint genes are mainly altered at the transcriptional level. Indeed, amplification and overexpression of *AURKA *(which encodes aurora-A kinase) have been observed in breast tumors and other cancers exhibiting aneuploidy [4]. *PLK1 *and *NEK2 *mRNA and protein expression is also elevated in a wide variety of tumors and cancer cell lines [5,6]. However, despite the importance of the mitotic spindle checkpoint in CIN, no detailed analyses of mitotic spindle checkpoint gene expression in tumors has yet been performed.

The recent development of effective tools for large-scale analysis of gene expression is providing new insights into the involvement of gene networks and regulatory pathways in various tumor processes [7]. It has also led to the discovery of new diagnostic and prognostic indicators, and to the identification of new molecular targets for drug development [8]. These tools include cDNA microarrays, which can be used to explore the expression of thousands of genes at a time, and real-time RT-PCR assays for more accurate quantitative studies of the expression of a smaller number of selected candidate genes.

As aneuploidy is common in breast cancer and is associated with a poor prognosis [9], we examined the expression of selected mitotic spindle checkpoint genes in breast tumors. We used real-time quantitative RT-PCR to measure the mRNA expression of a large number of selected genes in DNA aneuploid breast tumor samples, in comparison with DNA diploid breast tumor samples. We assessed the expression level of 76 genes known to be involved in various molecular mechanisms associated with the mitotic spindle checkpoint (Table 1). We identified nine genes involved in early breast tumorigenesis, and also a two-gene expression signature (*PLK1 *+ *AURKA*) associated with aneuploid status.

**Table 1:** List of the 76 selected genes

Gene symbols	Alternative symbols	Chromosome location	Genbank accession
**Mitotic spindle formation (n = 16)**			
***AURKA **^a^*	**Aurora-A, STK15, STK6**	**20q13.2-q13.3**	NM_003600
***AURKAIP1***	**AKIP**	**1p36.33**	NM_017900
***AURKB***	**Aurora-B; Aurora-1, STK12**	**17p13.1**	NM_004217
***AURKC***	**Aurora-C, STK13**	**19q13.43**	NM_003160
***BIRC5***	**Survivin**	**17q25**	NM_001168
***CDC20***	**Fizzy-R, fzy, p55CDC**	**1p34.1**	NM_001255
***CLASP1***		**2q14.2**	NM_015282
***CLASP2***		**3p22.3**	NM_015097
***FBXW7***	**AGO, hCDC4**	**4q31.3**	NM_033632
***FZR1***	**HCDH1**	**19p13.3**	NM_016263
***KNTC1***	**Rough Deal/ROD**	**12q24.31**	NM_014708
***RASSF1A***		**3p21.3**	NM_007182
***TPX2***	**C20orf**	**20q11.2**	NM_012112
***ZW10***	**Zeste-White**	**11q23.3**	NM_004724
***ZWILCH***	**FLJ10036**	**15q22.31**	NM_017975
***ZWINT***	**ZW10 interactor**	**10q21-q22**	NM_007057
***Centrosome cohesion and duplication *****(n = 2)**			
***CEP250***	**CEP2, C-NAP1**	**20q11.22**	NM_007186
***NEK2***	**NLK1**	**1q31.2-q41**	NM_002497
***Kinetochore-mitotic spindle interaction *****(n = 19)**			
***BUB1***		**2q14**	NM_004336
***BUB1B***	**BUBR1**	**15q15**	NM_001211
***BUB3***		**10q26**	NM_004725
***CENPE***	**CENP-E**	**4q24-q25**	NM_001813
***CSE1L***	**CAS**	**20q13**	NM_001316
***FBXO5***	**Emi1**	**6q25-q26**	NM_012177
***MAD1L1***	**MAD1**	**7p22**	NM_003550
***MAD2L1***	**MAD2**	**4q27**	NM_002358
***MAD2L2***	**REV7, MAD2B**	**1p36**	NM_006341
***NDC80***	**HEC1**	**18p11.31**	NM_006101
***PRCC***		**1q21.1**	NM_005973
***RAE1***		**20q13.31**	NM_003610
***RAN***		**12q24.3**	NM_006325
***RCC1***	**CHC1, RCC1**	**1p36.1**	NM_001269
***TACC1***		**8p11**	NM_006283
***TACC2***		**10q26**	NM_206862
***TACC3***		**4p16.3**	NM_006342
***TTK***	**MPS1 kinase**	**6q13-q21**	NM_003318
***UBD***	**FAT10**	**6p21.3**	NM_006398
***CDK-cyclin complexes *****(n = 7)**			
***CCNA1***	**Cyclin A1**	**13q12.3-q13**	NM_003914
***CCNA2***	**Cyclin A2**	**4q25-q31**	NM_001237
***CCNB1***	**Cyclin B1**	**5q12**	NM_031966
***CCNB2***	**Cyclin B2**	**15q21.2**	NM_004701
***CCNB3***	**Cyclin B3**	**Xp11**	NM_033031
***CDKN1A***	**p21(WAF1/CIP1)**	**6p21.2**	NM_000389
***CDC2***	**CDK1**	**10q21.1**	NM_001786
***Sister chromatid separation - mitotic exit *****(n = 29)**			
***ANAPC1***	**APC1**	**2q12.1**	NM_022662
***ANAPC10***	**APC10**	**4q31**	NM_014885
***ANAPC11***	**APC11**	**17q25.3**	NM_016476
***ANAPC2***	**APC2**	**9q34.3**	NM_013366
***ANAPC4***	**APC4**	**4p15.2**	NM_013367
***ANAPC5***	**APC5**	**12q24.31**	NM_016237
***ANAPC7***	**APC7**	**12q13.12**	NM_016238
***CDC16***	**APC6**	**13q34**	NM_003903
***CDC23***	**APC8**	**5q31**	NM_004661
***CDC26***		**9q32**	NM_139286
***CDC27***	**APC3**	**17q12-17q23.2**	NM_001256
***CDC34***		**19p13.3**	NM_004359
***ESPL1***	**Separase**	**12q13**	NM_012291
***HSPB1***	**HSP27**	**7q11.23**	NM_001540
***NEDD8***		**14q11.2**	NM_006156
***PLK1***	**Polo-like kinase 1**	**16p12.1**	NM_005030
***PPP1CA***	**PPP1A**	**11q13**	NM_002708
***PPP1R2***	**Inh2**	**3q29**	NM_006241
***PTTG1***	**Securin**	**5q35.1**	NM_004219
***RAD21***	**SCC1, KIAA0078**	**8q24**	NM_006265
***RNF2***	**Ding**	**1q25.3**	NM_007212
***SMC1A***	***SMC1L1***	**Xp11.22-p11.21**	NM_006306
***SMC1B***	***SMC1L2***	**22q13.31**	NM_148674
***SMC3***	**CSPG6**	**10q25**	NM_005445
***STAG1***	**SA1 (stromal antigen 1)**	**3q22.2**	NM_005862
***STAG2***	**SA2 (stromal antigen 2)**	**Xq25**	NM_006603
***UBE1C***	**UBA3**	**3p24.3-p13**	NM_003968
***UBE2B***	**UBE2B**	**5q23q-31**	NM_003337
***UBE2N***		**12q22**	NM_003348
***Double-strand break repair *****(n = 3)**			
***MRE11A***	**MRE11**	**11q21**	NM_005590
***BRCA1***		**17q21**	NM_007294
***BRCA2***		**13q12.3**	NM_000059

^a ^*Entrez Gene *symbol

## Results

### MRNA expression of 76 mitotic-spindle-checkpoint genes in invasive breast tumors relative to normal breast tissue

To select for further study those mitotic-spindle-checkpoint genes whose expression is dysregulated in breast tumors, we quantified the mRNA expression of the 76 selected genes in 10 invasive breast tumors relative to 5 normal breast tissues.

MRNA of all 76 genes was reliably quantifiable by means of real-time quantitative RT-PCR (Ct < 35) in both invasive breast tumors and normal breast tissues.

Forty (52.6%) of the 76 genes were significantly upregulated (P < 0.05) in the invasive breast tumors compared to the normal breast tissues (Table 2). The expression of 20 of these 40 upregulated genes was markedly higher (> 3-fold) in the breast tumors. The most strongly upregulated gene was *NEK2 *(29-fold).

**Table 2:** mRNA expression of 76 mitotic-spindle-checkpoint genes in invasive breast tumors relative to normal breast tissues

Genes	Normal breast tissues (n = 5)	Invasive breast tumors (n = 10)	p^a^
***NEK2***	**1.0 (0.44-2.23)**	**28.79 (7.41-162.02)^b^**	**< 0.01**
***UBD***	**1.0 (0.31-1.91)**	**16.95 (1.38-42.32)**	**< 0.01**
***TPX2***	**1.0 (0.59-1.48)**	**13.01 (5.51-144.34)**	**< 0.01**
***CENPE***	**1.0 (0.02-2.06)**	**11.01 (2.41-42.62)**	**< 0.01**
***CCNB2***	**1.0 (0.64-1.95)**	**10.36 (3.14-73.18)**	**< 0.01**
***BIRC5***	**1.0 (0.37-2.00)**	**9.45 (3.64-136.55)**	**< 0.01**
***NCD80***	**1.0 (0.29-1.23)**	**9.24 (2.08-114.83)**	**< 0.01**
***BUB1***	**1.0 (0.53-1.51)**	**8.52 (2.22-58.49)**	**< 0.01**
***CCNA2***	**1.0 (0.56-1.90)**	**8.08 (4.10-52.35)**	**< 0.01**
***CDC2***	**1.0 (0.68-1.66)**	**7.62 (2.59-44.74)**	**< 0.01**
***BUB1B***	**1.0 (0.53-1.69)**	**7.44 (2.47-35.02)**	**< 0.01**
***TTK***	**1.0 (0.71-2.08)**	**6.47 (1.27-36.76)**	**< 0.01**
***AURKB***	**1.0 (0.90-2.08)**	**5.56 (2.02-81.20)**	**< 0.01**
***PLK1***	**1.0 (0.60-1.87)**	**5.52 (2.72-44.53)**	**< 0.01**
***AURKA***	**1.0 (0.36-1.33)**	**4.76 (3.00-39.85)**	**< 0.01**
***TACC3***	**1.0 (0.54-2.08)**	**4.70 (1.73-21.06)**	**< 0.01**
***CCNB3***	**1.0 (0.96-4.20)**	**4.62 (0.80-39.31)**	**< 0.05**
***ZWINT***	**1.0 (0.62-1.97)**	**4.28 (1.78-21.76)**	**< 0.01**
***CCNB1***	**1.0 (0.42-2.32)**	**4.03 (1.10-15.63)**	**< 0.01**
***CDC20***	**1.0 (0.61-1.28)**	**3.51 (0.89-21.21)**	**< 0.05**
***PRCC***	1.0 (0.70-1.27)	2.70 (2.27-4.87)	**< 0.01**
***CDKN1A***	1.0 (0.61-2.57)	2.43 (1.04-5.59)	**< 0.05**
***RAN***	1.0 (0.59-1.92)	2.42 (1.23-6.57)	**< 0.01**
***ESPL1***	1.0 (0.34-1.85)	2.27 (1.23-8.79)	**< 0.05**
***PTTG1***	1.0 (0.82-1.35)	2.25 (1.61-11.24)	**< 0.01**
***KNTC1***	1.0 (0.71-1.30)	2.21 (0.80-4.83)	**< 0.05**
***BRCA2***	1.0 (0.70-1.41)	2.17 (0.68-5.86)	**< 0.05**
***RAE1***	1.0 (0.81-1.48)	2.16 (1.37-3.48)	**< 0.01**
***MAD2L1***	1.0 (0.65-1.30)	2.11 (1.16-5.25)	**< 0.01**
***AURKAIP1***	1.0 (0.94-1.59)	1.96 (1.30-4.68)	**< 0.01**
***PPP1CA***	1.0 (0.65-1.55)	1.95 (1.47-3.31)	**< 0.01**
***BUB3***	1.0 (0.65-1.20)	1.87 (1.27-5.64)	**< 0.01**
***ANAPC7***	1.0 (0.61-1.32)	1.77 (1.59-2.36)	**< 0.01**
***CDC27***	1.0 (0.57-1.36)	1.67 (1.19-2.32)	**< 0.01**
***ZWILCH***	1.0 (0.88-1.33)	1.63 (0.75-3.93)	**< 0.05**
***PPP1R2***	1.0 (067-1.18)	1.55 (0.81-2.06)	**< 0.05**
***MAD2L2***	1.0 (0.37-1.20)	1.45 (0.69-7.28)	**< 0.05**
***UBE1C***	1.0 (0.84-1.03)	1.40 (1.07-1.93)	**< 0.01**
***UBE2N***	1.0 (0.77-1.09)	1.31 (1.29-2.83)	**< 0.01**
***CDC23***	1.0 (0.71-1.18)	1.21 (0.79-1.53)	**< 0.05**
*SMC1B*	1.0 (0.43-1.81)	2.89 (0.07-10.65)	NS
*HSPB1*	1.0 (0.66-1.47)	2.03 (0.66-6.79)	NS
*TACC2*	1.0 (0.94-2.30)	1.70 (0.69-4.27)	NS
*ANAPC11*	1.0 (0.13-2.41)	1.65 (0.80-3.95)	NS
*CSE1L*	1.0 (0.61-1.23)	1.59 (0.75-3.87)	NS
*RAD21*	1.0 (0.71-1.17)	1.58 (0.57-8.38)	NS
*SMC3*	1.0 (0.71-1.45)	1.56 (0.59-3.26)	NS
*RCC1*	1.0 (0.39-1.68)	1.54 (0.87-3.75)	NS
*FBXO5*	1.0 (0.42-1.35)	1.50 (0.65-4.52)	NS
*BRCA1*	1.0 (0.75-1.29)	1.37 (0.58-5.67)	NS
*ANAPC10*	1.0 (0.56-1.65)	1.34 (0.88-1.75)	NS
*CEP250*	1.0 (0.87-1.54)	1.33 (0.88-3.08)	NS
*RNF2*	1.0 (0.96-1.13)	1.33 (0.64-2.88)	NS
*CDC34*	1.0 (0.33-1.52)	1.23 (0.63-1.97)	NS
*ANAPC1*	1.0 (0.75-1.43)	1.22 (0.54-1.59)	NS
*SMC1A*	1.0 (0.67-1.05)	1.09 (0.56-1.98)	NS
*UBE2B*	1.0 (0.72-1.77)	1.09 (0.41-2.11)	NS
*NEDD8*	1.0 (0.34-1.61)	1.08 (0.30-2.13)	NS
*ANAPC5*	1.0 (0.61-1.13)	1.07 (0.94-1.34)	NS
*ZW10*	1.0 (0.39-1.01)	1.07 (0.63-3.39)	NS
*STAG2*	1.0 (0.76-2.53)	1.05 (0.33-2.15)	NS
*CDC16*	1.0 (0.66-1.19)	0.99 (0.57-1.52)	NS
*CLAPS2*	1.0 (0.84-1.31)	0.98 (0.61-1.74)	NS
*CDC26*	1.0 (0.46-1.41)	0.97 (0.61-1.53)	NS
*CLASP1*	1.0 (0.84-1.54)	0.93 (0.71-1.45)	NS
*CCNA1*	1.0 (0.28-1.06)	0.84 (0.41-3.69)	NS
*MAD1L1*	1.0 (0.37-1.13)	0.69 (0.42-1.12)	NS
***TACC1***	1.0 (0.92-2.06)	0.78 (0.63-1.33)	**< 0.05**
***ANAPC2***	1.0 (0.40-1.23)	0.77 (0.62-1.45)	**< 0.05**
***FZR1***	1.0 (0.40-1.29)	0.73 (0.51-1.18)	**< 0.05**
***STAG1***	1.0 (0.68-1.14)	0.69 (0.36-1.00)	**< 0.05**
***ANAPC4***	1.0 (0.52-1.10)	0.68 (0.43-1.08)	**< 0.05**
***MRE11A***	1.0 (0.90-1.23)	0.64 (0.26-1.23)	**< 0.05**
***FBXW7***	1.0 (0.84-1.29)	0.56 (0.41-1.14)	**< 0.05**
***AURKC***	1.0 (0.63-1.43)	0.49 (0.34-2.66)	**< 0.05**
***RASSF1***	1.0 (0.17-3.10)	0.44 (0.12-1.68)	**< 0.05**

^a^Mann and Whitney's U Test

^b^Median (range) of gene mRNA levels. The mRNA levels of the tumor samples were normalized such that the median of the 5 normal breast tissues mRNA levels was 1.

In contrast, only 9 (11.8%) of the 76 genes were significantly down-regulated (P < 0.05) in the invasive breast tumors compared to the normal breast tissues, and none showed markedly lower expression (> 3-fold) in the breast tumors.

### Relationship between the mRNA expression of the 20 markedly upregulated genes and steps of breast tumor progression

To determine whether the 20 genes showing marked upregulation (> 3-fold) in the invasive breast tumors are altered at an early step of breast tumorigenicity, we analyzed their mRNA expression in 9 normal breast tissues, 14 benign breast tumors, 14 ductal carcinoma in situ (DCIS) of the breast, 11 invasive ductal grade I breast tumors and 12 invasive ductal grade III breast tumors (Table 3).

**Table 3:** Relationship between mRNA levels of 20 markedly upregulated genes and breast cancer progression

Genes	Normal breast tissues (n = 9)	Benign breast tumors (n = 14)	**p**^**a**^	DCIS of the breast (n = 14)	**p**^**b**^	Invasive grade I breast tumors (n = 11)	**p**^**c**^	Invasive grade III breast tumors (n = 12)	**p**^**d**^
***NDC80***	1,0 (0,00-1,39)	**3,64 (1,32-17,79)^e^**	**< 0,01**	**14,55 (4,10-24,20)**	**0,0009**	7,11 (1,38-13,55)	**NS**	**15,22 (4,76-54,69)**	**0,01**
***BUB1***	1,0 (0,00-1,38)	**2,97 (1,52-14,03)**	**< 0,01**	**15,37 (1,59-94,35)**	**0,001**	6,59 (0,00-94,35)	**NS**	**17,54 (5,46-57,28)**	**0,004**
***BUB1B***	1,0 (0,24-2,97)	**2,72 (1,01-9,38)**	**< 0,01**	**14,54 (3,53-39,95)**	**0,0002**	6,11 (0,00-11,31)	**NS**	**18,66 (2,39-105,05)**	**0,004**
***CCNB1***	1,0 (0,00-3,14)	**2,51 (1,01-6,82)**	**< 0,01**	**6,82 (1,71-15,24)**	**0,002**	4,23 (1,33-5,90)	**NS**	**9,05(3,51-41,64)**	**0,007**
***TACC3***	1,0 (0,00-1,35)	**1,70 (0,78-6,18)**	**< 0,01**	**7,21 (1,77-13,67)**	**0,0003**	**5,31 (1,11-11,24)**	**0,02**	**17,04 (4,98-74,03)**	**0,0006**
***TPX2***	1,0 (0,31-4,23)	**2,84 (0,82-10,73)**	**< 0,05**	**16,99 (4,70-35,59)**	**0,00009**	6,51 (1,67-19,74)	**NS**	**23,84 (6,15-315,17)**	**0,0009**
***CCNA2***	1,0 (0,05-1,45)	**2,19 (0,20-7,19)**	**< 0,05**	**10,56 (1,70-17,21)**	**0,0006**	3,36 (1,04-8,34)	**NS**	**11,38 (1,64-104,33)**	**0,008**
***CDC2***	1,0 (0,00-2,11)	**1,76 (0,76-7,36)**	**< 0,05**	**10,16 (2,56-20,87)**	**0,00008**	6,01 (1,06-10,78)	**NS**	**10,99 (3,32-56,75)**	**0,006**
***CDC20***	1,0 (0,06-1,28)	**1,67 (0,63-3,54)**	**< 0,05**	**3,90 (1,49-14,09)**	**0,0001**	1,65 (1,76-3,00)	**NS**	**6,14 (1,76-142,68)**	**0,0002**
***NEK2***	1,0 (0,16-2,87)	1,17 (0,41-3,78)	**NS**	**10,44 (2,03-26,23)**	**0,00004**	**2,67 (1,15-11,88)**	**0,008**	**14,83 (3,60-115,09)**	**0,0006**
***AURKA***	1,0 (0,30-2,58)	1,12 (0,33-2,32)	**NS**	**6,04 (1,27-21,01)**	**0,00002**	**2,54 (1,25-7,16)**	**0,002**	**7,82 (2,04-58,89)**	**0,003**
***PLK1***	1,0 (0,30-2,08)	0,80 (0,34-1,99)	**NS**	**3,83 (1,09-11,37)**	**0,00003**	**1,91 (0,20-5,66)**	**0,04**	**7,09 (1,92-117,27)**	**0,0009**
***TTK***	1,0 (0,01-7,32)	2,43 (0,00-11,29)	**NS**	**9,85 (2,59-32,07)**	**0,0003**	2,32 (0,00-5,68)	**NS**	**8,59 (4,14-55,84)**	**0,0002**
***AURKB***	1,0 (0,00-3,24)	2,07 (0,22-7,41)	**NS**	**5,97 (0,99-26,91)**	**0,02**	4,24 (1,05-10,06)	**NS**	**16,26 (5,70-210,84)**	**0,0003**
***BIRC5***	1,0 (0,46-3,40)	1,37 (0,39-6,06)	**NS**	**7,86 (1,68-40,50)**	**0,00008**	3,51 -0,54-7,29)	**NS**	**12,20 (3,14-128,0)**	**0,0007**
***ZWINT***	1,0 (0,00-3,71)	1,98 (0,39-4,53)	**NS**	**5,70 (2,45-15,74)**	**0,0002**	3,59 (0,89-6,09)	**NS**	**10,95 (2,70-55,46)**	**0,001**
***CCNB2***	1,0 (0,31-1,93)	1,43 (0,70-5,98)	**NS**	**8,98 (1,57-23,43)**	**0,0002**	3,31 (0,03-7,31)	**NS**	**12,28 (1,53-52,35)**	**0,005**
***CENPE***	1,0 (0,03-4,58)	0,96 (0,33-6,15)	**NS**	**3,61 (1,11-8,34)**	**0,002**	2,00 (0,33-5,57)	**NS**	**2,98 (0,84-10,15)**	**0,04**
***UBD***	1,0 (0,00-3,07)	1,49 (0,34-4,66)	**NS**	**3,19 (0,43-4,66)**	**0,04**	2,92 (0,19-14,72)	**NS**	5,30 (1,16-32,37)	**NS**
***CCNB3***	1,0 (0,00-6,11)	2,52 (0,53-5,82)	**NS**	1,36 (0,00-8,94)	**NS**	3,24 (0,63-8,54)	**NS**	2,71 (0,00-8,54)	**NS**
***MKI67***	1,0 (0,03-2,87)	2,63 (0,47-12,70)	**NS**	**14,92 (2,12-33,98)**	**0,0007**	5,21 (0,13-12,15)	**NS**	**14,09 (2,45-189,14)**	**0,009**

^a^Mann et Whitney's U Test: Benign breast tumors *vs *Normal breast tissues. NS, not significant.

^b^Ductal Carcinoma In Situ (DCIS) of the breast *vs *Benign breast tumors.

^c^Invasive grade I breast tumors *vs *Benign breast tumors.

^d^Invasive grade III breast tumors *vs *Invasive grade I breast tumors.

^e^Median (range) of gene mRNA levels. The mRNA levels of the tumor samples were normalized such that the median of the 9 normal breast tissues mRNA levels was 1.

The mRNA levels of 9 of the 20 selected genes (i.e. *NDC80, BUB1, BUB1B, CCNB1, TACC3, TPX2, CCNA2, CDC2 *and *CDC20*) was significantly increased in the benign breast tumors as compared to the normal breast tissues (Table 3). *NDC80 *was the gene with the strongest upregulation (3.6-fold).

With the exception of *CCNB3*, the expression of all 20 genes increased from benign breast tumors to DCIS.

Only *TACC3, NEK2, AURKA *and *PLK1 *expression increased from benign breast tumors to invasive ductal grade I breast tumors, while expression of all 20 genes (except *CCNB3 *and *UBD*) increased from grade I to ductal grade III breast tumors.

**Figure 1:**
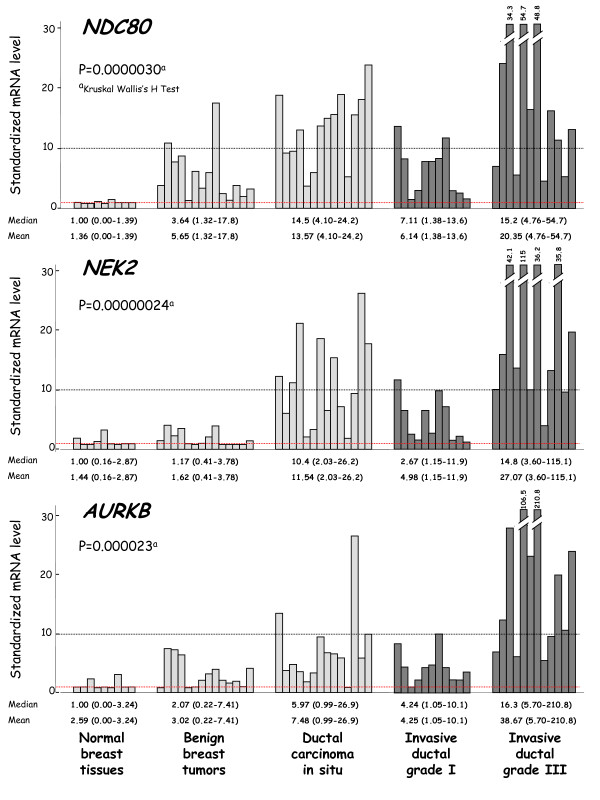
**mRNA levels of 3 characteristic upregulated genes (*NDC80, NEK2 *and *AURKA*) according to breast tumor progression**. Breast tumor progression groups are consisting of 9 normal breast tissues, 14 benign breast tumors, 14 ductal carcinoma in situ (DCIS), 11 invasive ductal grade I and 12 invasive ductal grade III breast tumors, respectively. Median values (ranges) and means +/- SD (in italics) are indicated for each tumor subgroup.

**Figure 2:**
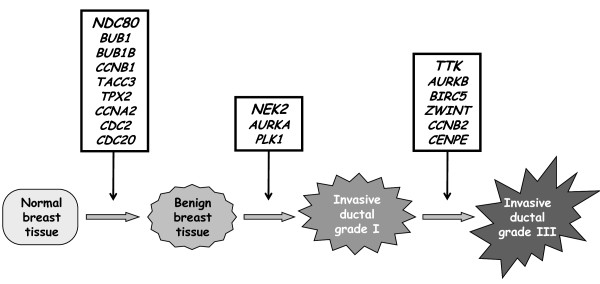
**Involvement of 18 characteristic genes in different steps of breast tumor progression**.

Figure 1 shows the mRNA levels of three characteristic genes (*NDC80, NEK2 *and *AURKB*) in the different sample types. Figure 2 shows the order in which these genes are dysregulated during the different steps of breast tumor progression.

In the same set of 60 samples, we also examined the expression of the proliferation-associated gene *MKI67*, which encodes the proliferation-related antigen Ki-67. *MKI67 *only showed significant overexpression in ductal carcinoma in situ (DCIS) and invasive ductal grade III breast tumors (Table 3).

### MRNA expression of the 20 markedly upregulated genes in breast cancer cell lines and in primary cultures of epithelial cells and fibroblasts from normal breast tissues and breast tumor cells

To determine in which tumor cell type (epithelial cells or stromal cells) the mitotic-spindle-checkpoint genes were upregulated, we measured the RNA levels of the 20 markedly upregulated genes in 12 breast cancer cell lines (five ERα-positive and seven ERα-negative cell lines). As compared to normal breast tissues, all 20 selected genes (except *UBD*) showed marked upregulation in the 12 breast cancer cell lines (median 3.9- to 87-fold), suggesting that these 19 genes are expressed in epithelial cells and upregulated in tumor epithelial cells (Table 4).

**Table 4:** mRNA expressions of the 20 markedly upregulated genes in breast cancer cell lines (ERa-negative and ERa-positive) and in primary cell cultures of epithelial cells and fibroblasts from normal breast tissues and breast tumor cells

Genes	Normal breast tissues (n = 9)	Breast tumor cell lines (n = 12)	ERα-negative cell lines (n = 7)	ERα-positive cell lines (n = 5)	p^a^	Normal fibroblasts	Normal epithelial cells	Tumoral fibroblasts	Tumoral epithelial cells
***AURKB***	1,0 (0,00-3,24)	**87,55 (20,39-163,71)^b^**	**118,19 (73,26-163,71)**	**32,90 (20,39-71,01)**	**< 0,01**	1,11	3,39	5,41	22,55
***TPX2***	1,0 (0,31-4,23)	**66,67 (23,37-123,35)**	**94,35 (59,99-123,35)**	**33,98 (23,37-47,50)**	**< 0,01**	1,88	2,33	6,76	18,38
***CDC20***	1,0 (0,06-1,28)	**25,90 (6,68-88,24)**	**38,68 (19,88-88,24)**	**10,22 (6,68-13,24)**	**< 0,01**	0,77	0,48	2,42	4,99
***BUB1***	1,0 (0,00-1,38)	**58,09 (16,34-155,96)**	**79,89 (39,81-155,96)**	**25,81 (16,34-45,41)**	**< 0,05**	2,76	1,64	6,66	28,54
***CCNA2***	1,0 (0,05-1,45)	**41,04 (9,75-79,34)**	**50,91 (18,64-79,34)**	**9,88 (9,75-38,05)**	**< 0,05**	1,39	2,04	4,63	13,18
***AURKA***	1,0 (0,30-2,58)	**40,98 (15,78-91,99)**	**50,68 (35,51-91,99)**	**20,49 (15,78-40,13)**	**< 0,05**	0,93	0,77	2,96	6,53
***CCNB1***	1,0 (0,00-3,14)	**37,66 (17,75-72,76)**	**55,91 (23,02-72,76)**	**23,26 (17,75-33,24)**	**< 0,05**	1,07	1,51	4,21	13,41
***BIRC5***	1,0 (0,46-3,40)	70,54 (28,64-179,15)	77,44 (58,69-179,15)	47,50 (28,64-146,52)	**NS**	1,25	2,23	6,00	14,98
***CCNB2***	1,0 (0,31-1,93)	41,50 (10,63-108,38)	47,34 (29,55-108,38)	19,97 (10,63-52,53)	**NS**	0,64	1,28	3,85	10,16
***BUB1B***	1,0 (0,24-2,97)	36,25 (13,18-94,35)	38,72 (30,91-94,35)	25,99 (13,18-45,10)	**NS**	0,98	1,37	4,48	17,33
***PLK1***	1,0 (0,30-2,08)	34,62 (9,99-50,45)	41,93 (22,68-50,45)	14,16 (9,99-37,10)	**NS**	0,55	0,56	2,62	4,73
***TACC3***	1,0 (0,00-1,35)	27,86 (9,94-69,07)	37,88 (12,24-69,07)	19,29 (9,94-33,75)	**NS**	1,82	1,52	3,15	7,50
***NDC80***	1,0 (0,00-1,39)	24,71 (5,70-209,38)	45,05 (5,70-209,38)	9,62 (6,92-16,37)	**NS**	1,60	1,10	6,45	17,23
***CDC2***	1,0 (0,00-2,11)	23,84 (6,26-76,46)	30,34 (8,86-76,46)	23,48 (6,26-13,24)	**NS**	0,90	0,49	4,13	11,99
***NEK2***	1,0 (0,16-2,87)	20,70 (5,45-62,25)	20,63 (6,53-62,25)	20,77 (5,45-40,32)	**NS**	1,71	0,94	4,12	4,37
***TTK***	1,0 (0,01-7,32)	17,95 (3,23-75,41)	25,75 (6,50-75,41)	12,73 (3,23-21,51)	**NS**	1,21	4,64	4,68	10,34
***ZWINT***	1,0 (0,00-3,71)	13,21 (3,97-34,14)	11,69 (3,97-34,14)	13,80 (4,32-17,51)	**NS**	0,60	0,62	1,64	4,03
***CENPE***	1,0 (0,03-4,58)	8,46 (0,33-14,83)	10,34 (0,87-12,27)	3,51 (0,33-14,83)	**NS**	1,09	0,83	1,78	10,08
***CCNB3***	1,0 (0,00-6,11)	3,87 (0,27-120,26)	3,35 (0,27-120,26)	4,39 (0,50-30,48)	**NS**	0,33	2,55	0,92	1,56
***UBD***	1,0 (0,00-3,07)	0,01 (0,00-11,39)	0,01 (0,00-0,13)	0,01 (0,00-11,39)	**NS**	0,06	2,56	0,26	0,49
***MKI67***	1,0 (0,03-5,99)	27,75 (7,38-54,07)	29,45 (13,36-54,07)	26,72 (7,38-31,78)	**NS**	0,95	0,86	3,99	16,00

^a^Mann and Whitney's U Test: ERa-positive cell lines vs ERa-negative cell lines. NS, not significant.

^b^Median (range) of gene mRNA levels. The mRNA levels of the tumor cell lines, fibroblasts and epithelial cells samples were normalized such that the median of the 9 normal breast tissues mRNA levels was 1.

Interestingly, the expression of these genes was generally higher in ERα-negative breast tumor cell lines than in ERα-positive lines. Despite the small number of cell lines analysed, seven genes (*AURKB, TPX2, CDC20, BUB1, CCNA2, AURKA*, and *CCNB1*) were upregulated significantly (p < 0.05) more strongly in the ERα-negative cell lines. These genes are probably not estrogen-regulated, but are rather upregulated mainly in undifferentiated breast tumors (i.e. ERα-negative tumors), independently of ERα status. Individual expression levels of these genes in the 12 breast tumor cell lines are shown in Additional File 1.

As tumors are composed not only of tumor epithelial cells but also of fibroblasts (the main cell type of the stromal compartment), we also measured the expression of the same 20 genes in primary cultures of epithelial cells and fibroblasts from normal breast tissues and breast tumor cells. We confirmed that these genes were expressed in epithelial cells and, to a lesser extent, in stromal fibroblasts, and that they were all upregulated in tumor epithelial cells, as compared to normal epithelial cells (Table 4).

### Relationship between the mRNA expression level and DNA amplification level of the 20 markedly upregulated genes

One of the 20 markedly upregulated genes (*AURKA*) has previously been shown to be upregulated by a DNA amplification mechanism [4]. Thus, to obtain further insight into the molecular mechanisms leading to overexpression of these 20 markedly upregulated genes, we used both real-time quantitative RT-PCR and high resolution array CGH to quantify the mRNA expression and DNA amplication of these genes in a series of 39 breast tumors (Table 5). Five of these genes (*NEK2*, *PLK, BIRC5, TPX2 *and *AURKA) *displayed DNA amplification (or polysomy) in more than 30% of breast tumors. Interesting, 3 of these 5 genes (*BIRC5, TPX2 *and *AURKA) *showed significantly higher mRNA levels in amplified tumors than in unamplified tumors. It is noteworthy that the other two genes (*NEK2 *and *PLK*), that showed similar mRNA levels in amplified and unamplified breast tumors, are located on chromosome arms (1q and 16p, respectively) showing polysomy and no DNA amplification in breast tumors [10,11].

**Table 5:** Relationship between the mRNA expression levels and DNA amplification levels of the 20 markedly upregulated genes

Genes	Chromosome location	Normal breast tissues (n = 6)	Breast tumors (n = 39)	Unamplified tumors		Amplified tumors		**p**^**a**^
						
				Number	mRNA level	Number	mRNA level	
***CDC20***	**1p34.1**	1,0 (0,61-1,28)	2,89 (0,39-22,65)^b^	38 (97,5%)	2,99 (0,54-22,65)	1 (2,5%)	0,39	NS
***NEK2***	**1q31,2-q41**	1,0 (0,44-5,23)	28,41 (2,46-137,03)	12 (30,8%)	26,55 (2,66-51,92)	**27 (69,2%)**	28,41 (2,46-137,03)	NS
***BUB1***	**2q14**	1,0 (0,53-1,51)	5,52 (0,74-25,63)	36 (92,3%)	5,43 (0,74-25,63)	3 (7,7%)	6,39 (3,46-17,15)	NS
***TACC3***	**4p16,3**	1,0 (0,54-2,08)	7,32 (1,01-29,89)	38 (97,5%)	7,09 (1,01-29,89)	1 (2,5%)	15,32	NS
***CENPE***	**4q21-q25**	1,0 (0,02-2,06)	14,59 (0,08-61,89)	38 (97,5%)	14,64 (0,08-61,89)	1 (2,5%)	5,54	NS
***CCNA2***	**4q25-q31**	1,0 (0,56-1,90)	10,39 (2,20-37,31)	36 (92,3%)	10,12 (2,2-37,31)	3 (7,7%)	22,65 (3,81-32,82)	NS
***CCNB1***	**5q12**	1,0 (0,42-2,32)	4,18 (0,34-22,47)	34 (87,2%)	3,53 (0,34-18,96)	5 (12,8%)	8,88 (2,67-22,47)	**0,01**
***UBD***	**6p21.3**	1,0 (0,31-3,91)	4,75 (0,15-106,40)	32 (82,1%)	4,89 (0,15-106,40)	7 (17,9%)	2,93 (0,54-7,43)	NS
***TTK***	**6q13-q21**	1,0 (0,71-2,08)	6,72 (0,61-44,27)	37 (94,9%)	6,72 (0,61-44,27)	2 (5,1%)	15,91 (5,85-25,96)	NS
***CDC2***	**10q21.1**	1,0 (0,68-2,66)	9,52 (1,19-56,17)	34 (87,2%)	8,44 (1,19-56,17)	5 (12,8%)	17,47 (12,74-42,86)	**0,03**
***ZWINT***	**10q21-q22**	1,0 (0,62-1,97)	5,38 (1,08-20,70)	32 (82,1%)	4,44 (1,08-20,70)	7 (17,9%)	12,52 (4,46-18,27)	**0,003**
***BUB1B***	**15q15**	1,0 (0,53-1,69)	10,48 (1,35-32,33)	34 (87,2%)	9,05 (1,35-32,33)	5 (12,8%)	17,33 (10,48-27,28)	**0,04**
***CCNB2***	**15q21.2**	1,0 (0,64-1,95)	14,21 (2,00-68,51)	35 (89,7%)	10,15 (2,0-55,08)	4 (10,2%)	21,41 (19,34-68,51)	**0,03**
***PLK***	**16p12.1**	1,0 (0,60-1,87)	5,46 (0,69-35,59)	16 (41,0%)	4,49 (0,69-21,61)	**23 (59,0%)**	5,46 (1,24-35,59)	NS
***AURKB***	**17p13.1**	1,0 (0,90-2,08)	5,82 (0,00-64,52)	39 (100%)	5,82 (0-64,52)	0	-	-
***BIRC5***	**17q25**	1,0 (0,37-2,0)	14,84 (1,47-150,30)	27 (69,2%)	9,96 (1,47-49,07)	**12 (30,8%)**	32,31 (5,84-150,30)	**0,0008**
***NDC80***	**18p11.31**	1,0 (0,29-1,23)	6,28 (1,05-126,38)	34 (87,2%)	5,69 (1,05-27,22)	5 (12,8%)	27,32 (4,13-126,38)	**0,03**
***TPX2***	**20q11.2**	1,0 (0,59-1,48)	15,69 (1,65-117,11)	22 (56,4%)	10,26 (1,65-34,46)	**17 (43,6%)**	24,03 (5,45-117,11)	**0,002**
***AURKA***	**20q13.2-q13.3**	1,0 (0,36-1,33)	7,14 (1,46-34,22)	24 (61,5%)	5,08 (1,46-32,82)	**15 (38,5%)**	14,04 (3,40-34,22)	**0,002**
***CCNB3***	**Xp11**	1,0 (0,96-4,20)	5,65 (0,00-61,53)	33 (84,6%)	4,76 (0,00-61,53)	6 (15,4%)	11,58 (5,16-25,25)	NS

^a^Mann and Whitney's U Test: amplified breast tumors vs unamplified breast tumors. NS, non significant.

^b^Median (range) of gene mRNA levels. The mRNA levels of the tumor samples were normalized such that the median of the 6 normal breast tissues mRNA level was 1.

### MRNA expression of the 49 dysregulated genes in 23 individual DNA aneuploid breast tumors and 24 DNA diploid breast tumors

The expression level of the 49 dysregulated genes identified in our screening study was then determined in a series of 23 DNA aneuploid breast tumors and 24 DNA diploid breast tumors (Table 6).

**Table 6:** Characteristics of the 24 DNA diploid and 23 DNA aneuploid breast tumors

	Human breast tumors (n = 47)		
	**DNA diploid breast tumors** ** (n = 24)**	**DNA aneuploid breast tumors** ** (n = 23)**	**P**^**a**^

*Age*			
≤ 05	2 (8.3%)	1 (4.3%)	NS
> 50	22 (91.7%)	22 (95.7%)	
*SBR histological grade *^b^			
I	9 (37.5%)	2 (8.7%)	**0.0061**
II	12 (50%)	9 (39.1%)	
III	3 (12.5%)	12 (52.2%)	
*Lymph node status*			
Negative	14 (58.3%)	15 (65.2%)	NS
Positive	10 (41.7%)	8 (34.8%)	
*Macroscopic tumor size*			
≤ 20 mm	12 (50%)	11 (48%)	NS
> 20 mm	12 (50%)	12 (52%)	
PR *status*			
Negative	3 (12.5%)	8 (34.8%)	NS
Positive	21 (87.5%)	15 (65.2%)	
ER *status*			
Negative	0 (0%)	7 (30.4%)	**0.012**
Positive	24 (100%)	16 (69.6%)	
ERBB2 *status*			
Negative	19 (79.2%)	17 (73.9%)	NS
Positive	5 (20.8%)	6 (26.1%)	
*Molecular subtypes*			
RH- ERBB2-	0 (0%)	4 (17.4%)	NS
RH- ERBB2+	0 (0%)	1 (4.4%)	
RH+ ERBB2-	19 (79.2%)	13 (56.5%)	
RH+ ERBB2+	5 (20.8%)	5 (21.7%)	
*Histologic types*			
Ductal	21 (87.5%)	23 (100%)	NS
Lobular	2 (8.3%)	0	
Tubular	1 (4.2%)	0	

^a ^Chi^2^ test.

^b^: Scarff Bloom Richardson classification.

Twenty-four (49.0%) of the 49 dysregulated genes were significantly upregulated in the 23 DNA aneuploid breast tumors relative to the DNA diploid breast tumors, while only one gene (*FZR1*) among the 49 dysregulated genes was significantly down-regulated (P < 0.05; Table 7).

**Table 7:** mRNA expression of the 49 dysregulated genes in aneuploid tumors relative to diploid tumors

Genes	Diploid tumors (n = 24)	Aneuploid tumor (n = 23)	**p***^**a**^*	**ROC-AUC**^**b**^
***PLK1***	**1,67 (0,69-7,46)^c^**	**8,65 (2,26-35,59)**	**0,0000005**	**0,929**
***AURKA***	**4,41 (1,46-15,80)**	**12,92 (3,88-34,22)**	**0,0000006**	**0,924**
***CCNB1***	**2,23 (0,34-19,16)**	**5,56 (2,67-18,97)**	**0,000002**	**0,901**
***BUB1***	**2,96 (0,74-10,74)**	**6,75 (1,88-25,63)**	**0,00008**	**0,837**
***CDC20***	**1,76 (0,39-7,95)**	**4,70 (1,39-22,65)**	**0,0001**	**0,824**
***TACC3***	**3,81 (1,01-12,27)**	**8,47 (2,62-29,89)**	**0,0002**	**0,814**
***CDC2***	**4,77 (1,19-23,21)**	**14,39 (3,51-56,17)**	**0,0005**	**0,797**
***ZWINT***	**3,74 (1,08-14,24)**	**5,97 (2,61-20,70)**	**0,0005**	**0,795**
***BUB3***	**1,73 (0,74-3,63)**	**2,89 (1,08-10,17)**	**0,0007**	**0,789**
***NDC80***	**4,30 (1,05-23,45)**	**10,00 (2,96-126,38)**	**0,0009**	**0,784**
***TPX2***	**9,63 (1,65-35,79)**	**22,11 (5,45-117,11)**	**0,0009**	**0,783**
***RAD21***	**1,61 (0,65-5,69)**	**3,03 (0,78-10,73)**	**0,001**	**0,770**
***MAD2L2***	**1,20 (0,39-3,66)**	**1,91 (0,61-7,41)**	**0,002**	**0,767**
***CDC23***	**1,27 (0,92-2,89)**	**1,96 (0,95-3,96)**	**0,002**	**0,765**
***PPP1R2***	**1,94 (0,83-3,46)**	**2,64 (1,03-4,91)**	**0,004**	**0,733**
***CENPE***	**7,58 (0,08-39,17)**	**17,11 (2,88-61,89)**	**0,007**	**0,729**
***PTTG1***	**2,60 (0,01-12,91)**	**4,03 (2,34-9,95)**	**0,007**	**0,729**
***BIRC5***	**9,76 (1,47-43,46)**	**23,21 (2,32-150,30)**	**0,007**	**0,728**
***AURKB***	**3,83 (0,61-20,14)**	**6,62 (1,02-64,52)**	**0,008**	**0,726**
***CCNB2***	**7,74 (2,00-68,51)**	**17,86 (3,53-55,08)**	**0,01**	**0,714**
***KNTC1***	**1,02 (0,44-3,63)**	**1,75 (0,50-3,70)**	**0,01**	**0,710**
***TTK***	**4,20 (0,61-16,04)**	**8,05 (1,72-44,27)**	**0,02**	**0,701**
***CCNA2***	**7,62 (2,45-32,82)**	**13,61 (2,20-35,30)**	**0,02**	**0,692**
***ESPL1***	**2,09 (0,44-9,64)**	**4,59 (0,68-10,11)**	**0,04**	**0,674**
***CDKN1A***	1,50 (0,49-3,87)	2,08 (0,76-10,29)	NS	0,657
***MAD2L1***	1,92 (0,80-8,58)	2,34 (1,08-5,18)	NS	0,647
***SMC1L2***	0,19 (0,00-2,15)	0,70 (0,04-10,43)	NS	0,642
***NEK2***	18,34 (2,46-109,01)	29,31 (4,97-137,03)	NS	0,638
***FBXW7***	0,47 (0,16-1,30)	0,61 (0,30-1,18)	NS	0,631
***BUB1B***	6,32 (1,35-63,78)	11,35 (3,25-27,28)	NS	0,626
***UBD***	3,47 (0,15-41,74)	4,94 (0,44-106,40)	NS	0,605
***CCNB3***	5,90 (0,75-27,35)	6,77 (1,29-61,53)	NS	0,601
***CDC27***	1,69 (0,74-2,46)	1,90 (0,65-5,77)	NS	0,599
***ANAPC4***	0,86 (0,31-1,84)	0,90 (0,38-1,90)	NS	0,592
***MRE11A***	0,72 (0,25-1,46)	0,89 (0,21-2,31)	NS	0,592
***ZWILCH***	1,79 (0,76-4,98)	2,26 (0,98-3,50)	NS	0,589
***BRCA2***	1,37 (0,31-7,84)	2,06 (0,69-4,87)	NS	0,581
***UBE2N***	2,08 (1,53-3,42)	2,34 (1,58-3,42)	NS	0,574
***RAN***	1,88 (1,05-3,33)	2,09 (1,28-15,85)	NS	0,550
***STAG1***	0,56 (0,23-1,03)	0,58 (0,31-0,94)	NS	0,549
***RAE1***	2,03 (1,37-4,32)	2,14 (1,15-2,88)	NS	0,519
***AURKC***	0,77 (0,37-3,08)	0,75 (0,35-10,67)	NS	0,496
***AURKAIP1***	2,00 (0,77-3,55)	1,83 (1,05-3,93)	NS	0,466
***PPP1CA***	2,57 (1,23-9,80)	2,15 (1,26-11,11)	NS	0,440
***TACC1***	1,14 (0,26-2,93)	0,99 (0,23-2,81)	NS	0,430
***RASSF1A***	0,55 (0,10-2,13)	0,46 (0,16-1,35)	NS	0,413
***ANAPC7***	2,32 (1,33-4,41)	2,22 (1,39-3,46)	NS	0,399
***PRCC***	3,67 (1,94-6,62)	2,96 (1,62-6,35)	NS	0,388
***UBE1C***	1,70 (0,67-3,71)	1,41 (0,52-2,65)	NS	0,384
***ANAPC2***	1,05 (0,46-2,63)	0,95 (0,57-1,62)	NS	0,365
***FZR1***	**0,75 (0,29-1,49)**	**0,56 (0,24-1,41)**	**0,001**	**0,225**
***HTERT***	**1,00 (0,51-4,11)**	**1,69 (0,33-28,84)**	**0,04**	**0,678**
***MKI67***	**1,00 (0,20-4,70)**	**2,20 (0,72-7,41)**	**0,0009**	**0,782**
***ESR1***	1,00 (0,29-2,78)	0,89 (0,00-3,77)	NS	0,426

^a^Kruskal Wallis' H Test

^b^ROC (Receiver Operating Characteristics) - AUC (Area Under the Curve) analysis

^c^Median (range) gene mRNA levels. The mRNA levels of the tumor samples were normalized such that the median of the 9 normal breast tissues mRNA levels was 1.

In the same set of 47 samples, we examined the expression of *MKI67 *and *ESR1/ERα*. As CIN of cancer cells could also be caused by telomere erosion [12], we examined the expression of the *TERT *gene encoding telomerase reverse transcriptase. *MKI67 *and *TERT *were significantly upregulated in the 23 DNA aneuploid breast tumors, while *ESR1/ERα *expression was similar in the diploid and aneuploid breast tumor subgroups (Table 7).

Prediction Analysis for Microarrays (PAM) and Class Prediction results obtained with the BRB Array Tools software packages were then used to identify a gene expression signature capable of discriminating between DNA aneuploid and DNA diploid breast tumors. Class Prediction identified a signature composed of 8 genes (*PLK1, AURKA, CCNB1, BUB1, TACC3, CDC20, CDC2 *and *TPX2*), while PAM identified a signature composed of only two genes (*PLK1 *and *AURKA*) that were also present in the Class Prediction signature.

**Figure 3:**
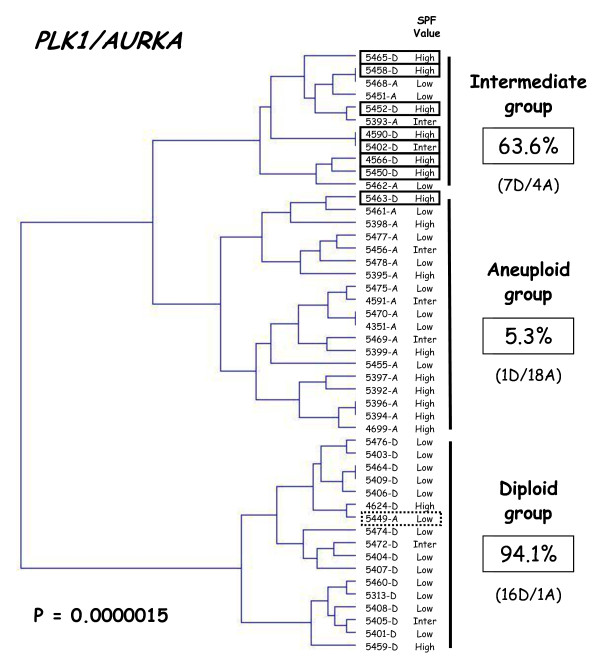
**Dendrogram of 24 DNA diploid (xxxx-D) and 23 DNA aneuploid breast tumors (xxxx-A)**. We constructed the dendogram by hierarchical clustering, according to PLK1 and AURKA expression. The SPF value, categorized as low, intermediate or high, is indicated for each tumor. The percentages of diploid breast tumors in each subgroup are indicated on the right.

Finally, hierarchical clustering of the 47 samples, based on *PLK1 *and *AURKA *expression, subdivided the patient population into three groups with significantly different ploidy (P = 0.0000015; figure 3), namely a DNA diploid group of 17 tumors (all but one showing DNA diploid status), an intermediate group of 11 tumors (7 DNA diploid and 4 DNA aneuploid) and a DNA aneuploid group of 19 tumors (all but one showing DNA aneuploid status). Interestingly, the SPF value of the DNA aneuploid tumor (5449-A; dotted line rectangle in figure 3) in the DNA diploid group was low, while the SPF values of the 8 DNA diploid tumors (solid line rectangles in figure 3) in the DNA aneuploid and intermediate groups were high (except for one with an intermediate SPF value).

### Validation of the two-gene expression signature in an independent series of breast tumor samples

**Figure 4:**
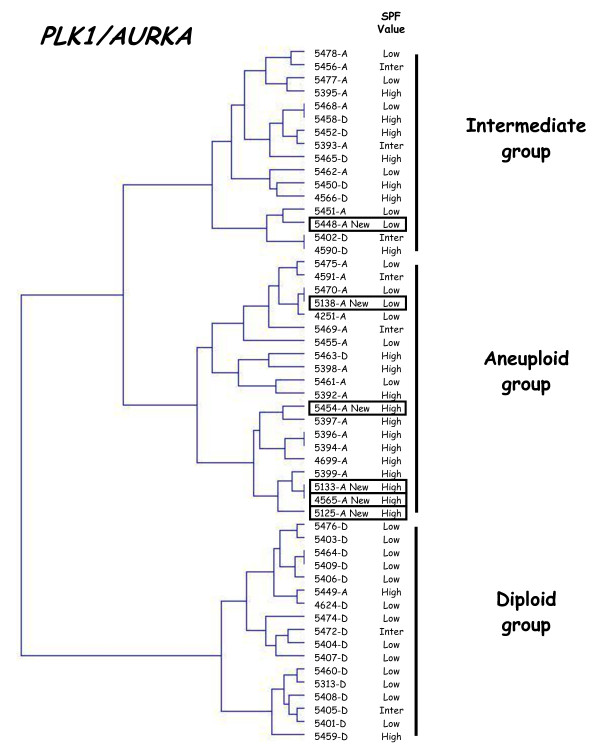
**Dendrogram of 24 DNA diploid, 23 DNA aneuploid and 6 additional DNA aneuploid breast tumors**. We constructed by hierarchical clustering, a dendrogram of 24 DNA diploid (xxxx-D), 23 DNA aneuploid (xxxx-A) and 6 additional DNA aneuploid breast tumors (xxxx-A-new; solid line rectangle), according to *PLK1 *and *AURKA *expression. The SPF value for each tumor, categorized as low, intermediate or high, is indicated on the right.

To validate our two-gene expression signature for tumor ploidy, we analyzed six additional classical DNA aneuploid breast tumors (1.10 ≤ ploidy index ≤ 1.90). All six tumors fell into the DNA aneuploid group (n = 5) or the intermediate group (n = 1) (figure 4). It is noteworthy that the DNA aneuploid tumor (5448-T) included in the intermediate group had a low SPF value.

**Figure 5:**
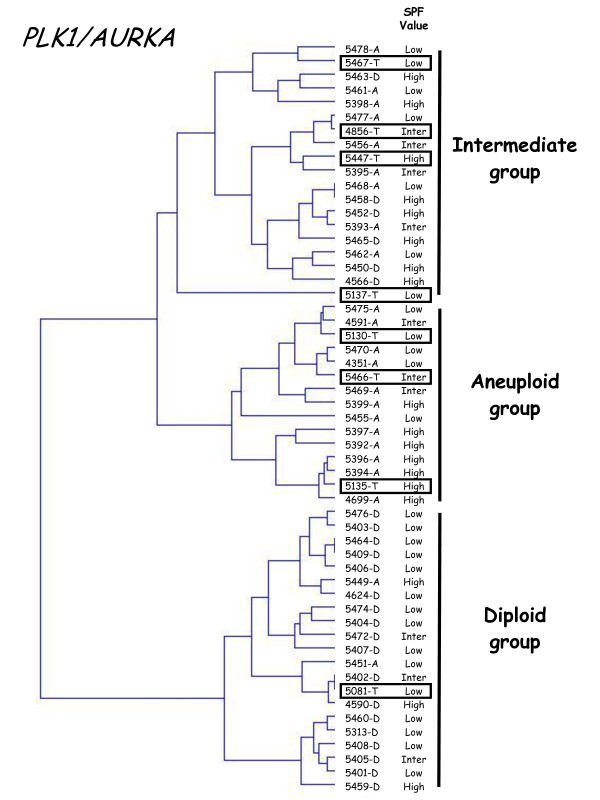
**Dendogram of 24 DNA diploid, 23 DNA aneuploid and 8 DNA tetraploid breast tumors**. We constructed by hierarchical clustering, a dendrogram of 24 DNA diploid (xxxx-D), 23 DNA aneuploid (xxxx-A) and 8 DNA tetraploid breast tumors (xxxx-T; solid line rectangle), according to PLK1 and AURKA expression. SPF value, categorized as low, intermediate or high, for each tumor is indicated on the right.

Recent studies suggest that abnormal division of tetraploid cells might facilitate genetic changes that give rise to aneuploid cancers and therefore that tetraploidy could be a transitional step between diploid status and classical aneuploid status [1]. Thus, we also analyzed 8 DNA tetraploid breast tumors (1.90 ≤ ploidy index ≤ 2.05) with our two-gene expression signature. All but one of these DNA tetraploid breast tumors fell into the DNA aneuploid group (n = 3) or the intermediate group (n = 4) (figure 5). It is noteworthy that the DNA tetraploid tumor (5081-T) included in the DNA diploid group had a low SPF value.

As the validation set includes a limited number of breast tumor samples, this two-gene expression signature capable of discriminating between DNA aneuploid and diploid breast tumors needs to be further validated in a large prospective randomized study.

## Discussion

To obtain further insight into the molecular mechanisms leading to aneuploidy in breast cancer, we used real-time quantitative RT-PCR to quantify the mRNA expression of a large number of selected genes in various types of breast tumor.

Real-time quantitative RT-PCR is a promising alternative to cDNA microarrays for molecular tumor profiling. In particular, real-time RT-PCR is far more precise, reproducible and quantitative than cDNA microarrays. Real-time RT-PCR is also more useful for analyzing weakly expressed genes, such as *TERT *in the present study. Finally, real-time RT-PCR requires smaller amounts of total RNA (about 2 ng per target gene), and is therefore suitable for analyzing small (benign or malignant) and microdissected tumor samples.

We studied a number of genes involved in various molecular mechanisms associated with the mitotic spindle checkpoint, and particularly genes already known to be altered (mainly at the transcriptional level) in various cancers [13-15]. These genes mainly encode proteins involved in mitotic spindle formation, centrosome cohesion and duplication, kinetochore-mitotic spindle interactions, CDK-cyclin complexes, and sister chromatid separation (see list in Table 1). This analysis was by no means exhaustive, and many possibly relevant genes were certainly missed, but it nevertheless demonstrates the ability of real-time RT-PCR to identify potentially useful marker genes.

Among the 76 genes analyzed, 49 (64.5%) showed significant dysregulation in breast tumors compared to normal breast tissues: 40 genes were upregulated (including 20 genes showing marked (> 3-fold) upregulation), while only nine genes were downregulated, and this downregulation was always moderate (< 3-fold) (Table 2).

To investigate if these genes are involved early in breast tumorigenesis (i.e. the transition from normal breast tissue to benign breast tumors and DCIS) or in tumor progression (i.e. the transition from invasive ductal grade I to invasive ductal grade III breast tumors), we studied the expression level of the 20 markedly upregulated genes in large panel of breast tissues, including normal breast tissues, benign breast tumors, DCIS, and grade I and III invasive ductal breast tumors (Table 3 and Figure 2).

Like *MKI67*, which encodes the proliferation-related antigen Ki-67, the expression of most of these genes (except *CCNB3 *and *UBD*) increased during the transition from grade I to ductal grade III breast tumors. Twelve genes (*NDC80, BUB1, CDC2, CCNA2, BUB1B, TACC3, TPX2, ZWINT, CCNB2, AURKB, NEK2 *and *BIRC5*) showed marked upregulation in ductal grade III breast tumors (more than 10-fold higher than in normal breast tissues), as well as in the breast tumor cell lines (up to 70-fold higher than in normal breast tissues). Most of these genes were specifically altered in tumor epithelial cells during malignant transformation.

These results are in total agreement with the literature showing a strong link between aneuploidy/CIN and tumor grade, i.e. between mitotic spindle checkpoint pathways and cell proliferation pathways. Indeed, several of the mitotic spindle checkpoint genes identified in this study (in particular *TPX2, NEK2, AURKA *and *PLK1*) have previously been included in a "proliferation signature" discriminating histological grades I and III [16], or in a "poor prognosis" signature [17,18].

These genes also showed marked upegulation in DCIS (higher than in ductal grade I breast tumors), confirming the major role of mitotic spindle checkpoint genes in pre-invasive lesions of the most common human cancers [19,20].

More interestingly, we identified 9 genes (*NDC80, BUB1, BUB1B, CCNB1, TACC3, TPX2, CCNA2, CDC2 and CDC20*) involved in the transition from normal breast tissues to benign breast tumors (Table 3). *NDC80/HEC1 *was the most strongly upregulated gene. Among the 14 benign breast tumors analyzed, 10 (71.4%) showed significant *NDC80/HEC1 *overexpression (> 3-fold higher than in normal breast tissues). *NDC80/HEC1 *is thus an outstanding candidate marker of breast lesions that are likely to undergo malignant transformation. *NDC80/HEC1 *regulates kinetochore microtubule dynamics and attachment stability [21]. Small molecule targeting Hec1 protein suppresses tumor cell growth in culture and in animal [22].

We identified a two-gene expression signature (*PLK1 *+ *AURKA*) associated with aneuploidy. *PLK1 *and *AURKA *are well-known mitotic spindle checkpoint genes that encode mitotic kinases (polo-like kinase-1 and aurora A, respectively). These enzymes are emerging as critical regulators of centrosome cycling and formation of the bipolar mitotic spindle [23-25]. These two genes are overexpressed in many types of solid tumor. *AURKA *lies within a region of human chromosome arm 20q13 that is amplified in breast cancer [4], as confirmed here (Table 5). Further in vitro studies (cultured cells) and in vivo studies (animal models) will be required for full confirmation of the role of these two genes in the molecular mechanisms leading to breast cancer aneuploidy.

Based on our two-gene expression signature, we subdivided the patient population (n = 47) into three groups with significantly different ploidy, namely a DNA diploid group (n = 17), a DNA aneuploid group (n = 19), and an intermediate group (n = 11) including both DNA aneuploid and DNA diploid tumors (figure 3). Interestingly, the SPF values of all the DNA diploid tumors in the intermediate group were high, confirming the relationship between aneuploidy and proliferation. A large prospective randomized study will be necessary to confirm the existence of this intermediate group and to determine the diagnostic and prognostic relevance of these 3 subgroups.

It is also noteworthy that the expression of the *TERT *gene, encoding telomerase reverse transcriptase, was significantly upregulated in DNA aneuploid breast tumors compared to DNA diploid breast tumors, confirming that aneuploidy may also be caused by telomere erosion [12].

Based on this two-gene expression signature, some DNA tetraploid tumor samples failed to cluster in the DNA aneuploid breast tumor group, in keeping with the observation that aneuploidy can be preceded by tetraploidy [26].

In conclusion, this study confirms the strong relationship between aneuploidy and proliferation. Among a panel of 76 mitotic spindle checkpoint genes, we identified several genes of interest whose expression status might serve to guide individual breast cancer patient management. Some of the genes identified here are already used to predict tumor recurrence and the response to treatment, while *AURKA *and *PLK1 *are frequently included in "poor prognosis" signatures [17,18,27]. Multivariate analyses will be necessary to assess the potential of our 2-gene signature as comparated to existing gene-expression signatures such as Mammaprint^® ^and Oncotype DX^®^, and a already identified gene expression signature of genomic instability to improve grading of breast tumors [28] or to predict the clinical outcome of breast cancer patients [29]. *AURKA *amplification induces resistance to taxol [30] and several aurora kinase inhibitors and polo-like kinase 1 inhibitors are in the preclinical development phase [6,31-33]. Finally, the finding that *NDC80/HEC1 *is involved early in breast carcinogenesis suggests that it too may have clinical relevance.

## Materials and methods

### Patients and Samples

To characterize gene expression signatures associated with breast tumor ploidy, we analyzed samples of 47 primary breast tumors (23 DNA aneuploid and 24 DNA diploid tumors) excised from women at our institution. Samples containing more than 70% of tumor cells were considered suitable for this study. Tumor cellularity was assessed on hematoxylin and eosin-stained tissue sections. Immediately after surgery the tumor samples were The samples were placed in liquid nitrogen until RNA extraction.

The patients met the following criteria: primary unilateral non metastatic breast carcinoma; complete clinical, histological and biological information available; no radiotherapy or chemotherapy before surgery; and full follow-up at our institution.

Estrogen receptor, progesterone receptor and ERBB2 status was determined at the protein level by biochemical methods (dextran-coated charcoal method, enzymatic immuno-assay or immunohistochemistry) and confirmed by ERα, PR and ERBB2 real-time quantitative RT-PCR assays. Using RH (ERα and PR) and ERBB2 status, we subdivided the total population (n = 47) into 4 subgroups, i.e. HR+ (ER+ and/or PR+)/ERBB2+ (n = 10), HR+ (ER+ and/or PR+)/ERBB2- (n = 32), HR- (ER- and PR-)/ERBB2+ (n = 1), and HR- (ER- and PR-)/ERBB2- (n = 4).

Standard prognostic factors are shown in Table 6. The median follow-up was 7,8 years (range 26 months to 11.25 years).

The patients had physical examinations and routine chest radiography every 3 months for 2 years, then annually. Mammograms were done annually.

To validate and explore our gene expression signature associated with tumor ploidy, we analyzed 14 additional DNA aneuploid breast tumors, comprising 6 classical aneuploid and 8 DNA tetraploid breast tumor.

To investigate the relationship between the mRNA levels of candidate genes and breast cancer progression, we also analyzed the RNA of 14 benign breast tumors, 14 ductal carcinoma in situ (DCIS) of the breast, 11 invasive ductal grade I breast tumors, and 12 invasive ductal grade III breast tumors. Standard prognostic factors for the 11 invasive ductal grade I breast tumors and 12 invasive ductal grade III breast tumors are indicated in Additional File 2, along with standard prognostic factors for the 10 invasive breast tumors used for initial screening of the dysregulated genes.

Patients' consent and approval from the Local Ethical Committee (Breast Group of René Huguenin Hospital) was obtained prior to the use of these clinical materials for research purposes in agreement to the Declaration of Helsinki. The biological collection has been recorded at the French Ministry of Research (N° DC-2008-355).

Finally, we analyzed five ERα-positive cell lines (MCF7, HCC1500, T-47D, ZR-75-1 and MDA-MB361) and seven ERα-negative cell lines (SK-BR-3, HBL-100, BT-20, MDA-MB157, MDA-MB231, MDA-MB435s and MDA-MB468), obtained from the American Tissue Type Culture Collection.

Nine specimens of adjacent normal breast tissue from breast cancer patients or normal breast tissue from women undergoing cosmetic breast surgery were used as sources of normal RNA.

### Primary cell culture and differential isolation of epithelial cells and fibroblasts from normal breast tissues and breast tumor cells

To determine which cells (epithelial cells and/or fibroblasts) overexpressed mitotic-spindle-checkpoint genes, we measured the RNA levels of the selected genes in primary cultures of epithelial cells and fibroblasts from normal breast tissues and breast tumor cells.

Breast tumors and normal tissues were minced with a scalpel and incubated overnight with Liberase Blendzyme 2 (Roche Applied Science, Meylan, France) for enzymatic dispersion. Organoids and aggregated cells (epithelial fraction) and isolated cells (fibroblast fraction) were separated by filtration and centrifugation. The fibroblast fraction was cultured in Ham's F10 medium containing L-glutamine (3 mM), insulin (5 mg/mL), T3 (1 nM), hydrocortisone (1 mg/mL), kanamycin (0.1 mg/mL), and 10% fetal calf serum. The epithelial fraction was cultured in the same conditions, plus epidermal growth factor (5 ng/mL), transferrin (5 mg/mL) and 5% human serum (instead of fetal calf serum). Cells were incubated in humidified air with 5% CO2 at 37°C, and the medium was changed three times a week. Cells were cultured for two weeks before RNA extraction. Epithelial cells and fibroblasts were identified by their morphological features and by detecting epithelial (keratin 19) and fibroblast marker expression with real-time RT-PCR.

### Flow cytometric DNA analysis and S-phase fraction (SPF) classification

Cell preparation and DNA staining were performed as previously described [34]. Flow cytometry (FCM) was performed on a FACScalibur device (Becton Dickinson, CA, USA). Cell cycle analysis was performed with the Modfit LT 2.0 program (Verity Software House, Topsham, ME). The DNA-diploid peak was located on DNA histograms by using an external standardization procedure with normal human lymphocytes positioned in the fifth part of the red fluorescence scale. DNA ploidy and the S-phase fraction (SPF) were obtained after gating on a dot plot (FL2-width versus FL2-area), selecting a representative amount of debris and excluding doublets.

The DNA ploidy pattern was expressed as the DNA index (DI) that is the ratio between the mean fluorescence channel number of the tumor G0/G1 peak and the diploid G0/G1 reference peak. Rules established during a previous inter-laboratory control procedure [35] were applied when using the cell-cycle software models. The tumors were classified as follows based on the DNA index. A tumor showing a single peak with a DNA index comprised between 0.95 and 1.1 was classified as DNA diploid; if an additional peak was present, the tumor was placed in one of the following DNA aneuploid subcategories, if they contain at least 10% of total cell counts and a corresponding G2M peak: DNA aneuploid with a DI comprised between 1.10 and 1.90 and > 2.05; DNA tetraploid with a DI comprised between 1.90 and 2.05. There were no hypodiploid (DI < 0.95) or multiploid (several aneuploid peaks) tumors in this series. The ploidy-adjusted SPF was categorized as low, intermediate or high, based on the 33rd and 66th percentiles. The debris and aggregate subtraction options were used when appropriate.

### Real-time RT-PCR

#### (1) RNA extraction

Total RNA was extracted from breast specimens by using the acid-phenol guanidium method. The quantity of the RNA samples was accurately measured by using a NanoDrop spectrophotometer, and their quality was determined by electrophoresis through agarose gel staining with ethidium bromide, and visualization of the 18S and 28S RNA bands under ultraviolet light."

#### (2) Theoretical basis

Real-time PCR reactions are characterized by the point during cycling when amplification of the PCR product is first detected, rather than the amount of PCR product accumulated after a fixed number of cycles. The parameter Ct (threshold cycle) is defined as the fractional cycle number at which the fluorescence generated by cleavage of a TaqMan probe (or by SYBR green dye-amplicon complex formation) passes a fixed threshold above baseline. The increase in the fluorescence signal associated with exponential growth of PCR products is detected by the laser detector of the ABI Prism 7700 Sequence Detection System (Perkin-Elmer Applied Biosystems, Foster City, CA), using PE Biosystems analysis software, according to the manufacturer's manuals.

The precise amount of total RNA added to each reaction mix (based on optical density) and its quality (i.e. lack of extensive degradation) are both difficult to assess. We therefore also quantified transcripts of two endogenous RNA control genes involved in two cellular metabolic pathways, namely *TBP *(Genbank accession NM_003194), which encodes the TATA box-binding protein, and *RPLP0 *(NM_001002), which encodes human acidic ribosomal phosphoprotein P0. Each sample was normalized on the basis of its *TBP *(or *RPLPO*) content.

Results, expressed as N-fold differences in target gene expression relative to the *TBP *(or *RPLPO*) gene, and termed "N*target*", were determined as N*target *= 2^ΔCt*sample*^, where the ΔCt value of the sample is determined by subtracting the average Ct value of the target gene from the average Ct value of the *TBP (or RPLP0) *gene [36,37].

The N*target *values of the samples were subsequently normalized such that the median of the nine normal breast tissue N*target *values was 1.

#### (3) Primers and controls

Primers for *TBP, RPLP0 *and the 76 target genes were chosen with the assistance of the Oligo 5.0 computer program (National Biosciences, Plymouth, MN).

To avoid amplification of contaminating genomic DNA, one of the two primers was placed at the junction between two exons. In general, amplicons were between 70 and 120 nucleotides long. Gel electrophoresis was used to verify the specificity of PCR amplicons.

The 76 target genes tested in this study are listed in Table 1. They were selected from the literature for their potential involvement in molecular mechanisms associated with the mitotic spindle checkpoint.

cDNA synthesis and PCR conditions were as described elsewhere [37]. Experiments were performed with duplicates for each data point. All patient samples with a CV of Ct values higher than 10% were retested.

### High-resolution array CGH (comparative genomic hybridization)

Tumor samples were analyzed with the Agilent Human Genome CGH Microarray 44K. DNA samples for array CGH were labeled as previously described [38]. Briefly, 1 μg each of breast tumor DNA and commercial pooled human normal genomic DNAs (Promega, Madison, WI) was digested with 5 μg of AluI (50 units) and 5 ml of RsaI (50 units) (Promega, Madison, WI) and labeled by random priming with CY3- and CY5-dUTP, respectively (Agilent Technologies, Massy, France). The labeled solutions were then filtered on a Microcon YLM-30 column (Millipore, Billerica, MA), denatured and hybridized with unlabeled Cot-1 DNA (Invitrogen, Carlsbad, CA) to the CGH arrays. After hybridization in an oven rotating at 15 rpm (Model1012, Sheldon Manufacturing, Cornelius, OR), the slides were washed and scanned with the Agilent G2565AA Microarray Scanner.

### Statistical Analysis

As the mRNA levels did not fit a Gaussian distribution, (a) the mRNA levels in each subgroup of samples were expressed as the median and range rather than the mean and coefficient of variation, and (b) relationships between the molecular markers and clinical and histological parameters were analyzed with the chi-square test (link between two qualitative parameters) or the non parametric Mann-Whitney *U *test (link between one qualitative parameter and one quantitative parameter) [39]. Differences between two populations were considered significant at confidence levels greater than 95% (p < 0.05).

To visualize the capacity of a given molecular marker to discriminate between two populations (in the absence of an arbitrary cutoff value), we summarized the data in a ROC (receiver operating characteristics) curve [40]. ROC curves plot sensitivity (true positives) on the *Y *axis against 1-specificity (false positives) on the *X *axis, considering each value as a possible cutoff. The AUC (area under curve) was calculated as a single measure of the discriminatory capacity of each molecular marker. When a molecular marker has no discriminatory value, the ROC curve lies close to the diagonal and the AUC is close to 0.5. In contrast, when a molecular marker has strong discriminatory value, the ROC curve moves to the upper left-hand corner and the AUC is close to 1.0.

A gene expression signature associated with tumor ploidy was sought with the BRB Array Tools program, using the *Prediction Analysis for Microarrays (PAM) *and *Class Prediction results *modules.

Hierarchical clustering was performed with GenANOVA software [41].

## Abbreviations

CGH: Comparative genomic hybridization; CIN: Chromosomal instability; Ct: Cycle threshold; DCIS: Ductal carcinoma in situ; FCM: Flow cytometry; RT-PCR: Reverse transcriptase-polymerase chain reaction; SPF: S-phase fraction.

## Competing interests

The authors declare that they have no competing interests.

## Authors' contributions

IB, SV, HB and STK carried out real-time RT-PCR study and analysis. FLa and KD performed cell culture and isolation of cells. MB and FS performed flow cytometric DNA analysis. ER and STK performed the CGH-array study and analysis. HR and GCC performed the statistical analysis. IB and RL conceived the study and participated in its design and coordination. IB, RL, FS and FLe drafted the manuscript. All authors read and approved the final manuscript.

## Supplementary Material

Additional file 1 **mRNA levels of the 20 marked upregulated genes in ERa-negative and ERa-positive breast cancer cell lines**. Click here for file

Additional file 2 **Characteristics of the 33 breast tumors (10 for pre-screnning, 11 invasive grade I and 12 invasive grade III)**. Click here for file
