# Supplementary material for: Expression analysis of mitotic spindle checkpoint genes in breast carcinoma: role of NDC80/HEC1 in early breast tumorigenicity, and a two-gene signature for aneuploidy
Source: Mol Cancer. 2011 Feb 27;10:23. doi: 10.1186/1476-4598-10-23 (PMC3058099; doi:10.1186/1476-4598-10-23)
Supplement: Additional file 1 — mRNA levels of the 20 marked upregulated genes in ERa-negative and ERa-positive breast cancer cell lines. [file 1476-4598-10-23-S1.DOC]

**Additional file 1.** mRNA levels of the 20 marked upregulated genes in ERa-negative and ERa-positive breast cancer cell lines.

The mRNA levels of the tumor cell lines were normalized such that the median of the 9 normal breast tissues mRNA levels was 1.
