# Supplementary material for: Expression analysis of mitotic spindle checkpoint genes in breast carcinoma: role of NDC80/HEC1 in early breast tumorigenicity, and a two-gene signature for aneuploidy
Source: Mol Cancer. 2011 Feb 27;10:23. doi: 10.1186/1476-4598-10-23 (PMC3058099; doi:10.1186/1476-4598-10-23)
Supplement: Additional file 2 — Characteristics of the 33 breast tumors (10 for pre-screnning, 11 invasive grade I and 12 invasive grade III). [file 1476-4598-10-23-S2.DOC]

**Additional file 2**. Characteristics of the 33 breast tumors (10 for pre-screnning, 11 invasive grade I and 12 invasive grade III).

|  | **Human breast tumors (n= 33)** | | |
| --- | --- | --- | --- |
|  | Pre-screening breast tumors (n=10) | breast tumors (n=23) | P^a^ |
| *Age*  ≤50  >50 | **0**  **10** | **3**  **20** | **NS** |
| *SBR histological grade* b  I  II  III | **3**  **3**  **4** | **11**  **0**  **12** | **0.022** |
| *Lymph node status*  Negative  Positive | **7**  **3** | **16**  **7** | **NS** |
| *Macroscopic tumor size*  ≤20mm  >20mm | **6**  **4** | **18**  **5** | **NS** |
| PR *status*  Negative  Positive | **5**  **5** | **8**  **15** | **NS** |
| ER *status*  Negative  Positive | **4**  **6** | **7**  **16** | **NS** |
| ERBB2 status  Negative  Positive | **7**  **3** | **18**  **5** | **NS** |
| *Histologic type*  Ductal  Lobular  Tubular | **9**  **0**  **1** | **22**  **0**  **1** | **NS** |

a: χ2 Test.

b: Scarff Bloom Richardson classification.
